# An observational cohort study of the effects of septoplasty with or without inferior turbinate reduction in patients with obstructive sleep apnea

**DOI:** 10.1186/1472-6815-14-11

**Published:** 2014-10-21

**Authors:** Mads Henrik Strand Moxness, Ståle Nordgård

**Affiliations:** 1 Center for Endoscopic Nasal and Sinus surgery, Aleris Hospital Trondheim, Trondheim, Norway; 2 The department of Otorhinolaryngology, Head and Neck Surgery, St Olav University Hospital, Trondheim, Norway; 3 The Institute of Neuroscience, The Norwegian University of Science and Technology (NTNU), Trondheim, Norway; 4 Post: Department of Neuroscience, NTNU, The Medical Faculty, N-7489 Trondheim, Norway

**Keywords:** Apnea, Nose, Surgery, Septum, Concha, Turbinate

## Abstract

**Background:**

The objective of this observational study was to evaluate the outcomes of intranasal surgery in patients with obstructive sleep apnea (OSA) in a single institution in Norway.

**Methods:**

Fifty-nine patients with OSA and clinically significant nasal obstruction underwent either septoplasty alone or septoplasty with concomitant volume reduction of the turbinates from August 2008 until the end of December 2010. Subjects were scheduled for sleep polygraphy before and 3 months after treatment.

In this observational single-centre cohort study we evaluated and compared the effect of these two specific surgical procedures on sleep related parameters.

**Results:**

There was a significant reduction in the apnea-hypopnea index (AHI) only in the group that had septoplasty with turbinate reduction (17.4, (SD 14.4) – 11.7, (SD 8.2), p <0.01), and this effect was significantly better than in the group treated with septoplasty alone. Other objective parameters remained unchanged. Subjective assessments obtained with a postoperative questionnaire showed an equally positive effect on diurnal sleepiness and nasal obstruction in both groups, and a better effect on sleep quality in the combined treatment group.

**Conclusion:**

The effect of nasal surgery on obstructive sleep apnea seemed to be greater when there were indications for combined surgery of the inferior turbinates and the nasal septum, compared to when there were indications for septoplasty alone.

## Background

There is growing interest in the field of sleep-related disorders (SRD) and in obstructive sleep apnea (OSA) particularly. This is due to the impact of SRD on global health, and a result of more profound insight into the effects of sleep deprivation, and the biomechanical and physiological changes that occur during the development of upper airway collapse during sleep [1]. The traditional way of understanding the collapsing airway includes both theories of neuromuscular regulation [2] and theories of fluid structure interaction [3]. Surgical treatments for OSA have been performed in several forms over the last 3 decades [4]. To date, tracheotomy is the only surgical procedure with definite and lasting success, but it is regarded as a method with unwanted side effects. Multiple level surgery has gained support, as well as maxillomandibular surgery, but these are also major procedures and the same concerns regarding morbidity apply for these. The effect of limited and less extensive surgery of the upper airways still needs evaluation regarding selection of procedure and results. Nasal surgery has been performed extensively in these patients, often with good effect on quality of life (QOL) measures [5,6]. Still, there is no conclusive evidence of clinical effect, and the different nasal procedures performed are often quite randomly chosen. To our knowledge, there are no other clinical studies that compare the results of different nasal procedures for nasal obstruction in patients with OSA. We have evaluated and compared the results of two specific surgical procedures in the nasal cavity, septoplasty alone and septoplasty with simultaneous turbinate volume reduction.

## Methods

This study was an observational single-centre cohort study. It was approved by the national regional ethics committee and was registered in Clincaltrials.gov. (NCT01282125). Between August 2008 and December 2010, 78 patients with OSA were treated surgically for nasal obstruction in Aleris Hospital in Trondheim, Norway. Fifty-nine of these had been treated with septoplasty alone or septoplasty combined with volume reductive surgery of the turbinates. Group 1 (n = 33) consisted of patients who had undergone septoplasty alone, and group 2 (n = 26) of patients treated with combined septoplasty and volume reductive surgery. The remaining patients underwent rhinoseptoplasties (n = 8), functional endoscopic sinus surgery (n = 4) and turbinate resection (n = 7) as single procedures, but the groups were too small to be subanalyzed. All patients in the two analyzed groups underwent traditional cartilage preserving septoplasty under general anesthesia. The volume reductive surgery comprised radiofrequency tissue ablation (n = 10) (BM 780-II, Sutter Medizintechnik Gmbh), lateral fracture of the lower turbinate (n = 15), and surgical reduction of concha bullosa (n = 1).

The patients were referred to the sleep clinic for suspected OSA from either primary care physicians or ENT specialists within a specific geographical area. All patients underwent a nocturnal sleep evaluation with an Embletta™ Portable Diagnostic System (ResMed, San Diego, California, USA) or a Reggie polygraph (Camtech, Oslo, Norway) and a clinical examination. There were no prior history of nasal surgery or prolonged use of nasal steroids. None of the patients were diagnosed with chronic rhinosinusitis or enlarged adenoids. Patients with confirmed OSA and clinically significant nasal obstruction due to a septal deviation with or without hypertrophy of turbinates were offered intranasal surgery as a first line of treatment. The decision to supplement septoplasty with volume reductive surgery in selected patients was based on the clinical evaluation, and not supported by objective measurements. If there were a coherence between the patients complaints of nasal blockage on both sides, and there was obvious swelling of the inferior turbinates that was relieved after decongestion with tetracain/adrenalin over 5-10 minutes in the office, one would recommend that turbinate reduction should be performed at the time of the septal surgery. Only patients with apnea-hypopnea index (AHI) >5 and BMI <35 were included. All patients used saline irrigation 6-8 times a day for two weeks postoperatively. No intranasal steroids were administered. Optional pain relief was 50 mg of diclofenac sodium three times a day and 30-60 mg of codein phosphate in combination with 500 mg of paracetamol. The same surgeon (MM) treated all but one patient. The patients were informed of the possibility of crusting in the nose for a period up to three weeks after surgery, but there were no postoperative infections and no necrosis or loss of nasal function at the follow up three months later.

The effects of intranasal surgery on OSA were evaluated routinely after 3 months with a repeated polygraph. Subjective assessment of daytime sleepiness was evaluated using the Epworth Sleepiness Scale (ESS) preoperatively and 3 months postoperatively. In a dichotomous questionnaire, the patients were asked to evaluate the effects of surgery on nasal obstruction and the subjective quality of sleep. At the same time a written informed consent was obtained from all the participants. The alternatives in the questionnaire were: 1. Did you experience an effect on your nasal obstruction after surgery? Yes or No. 2. Did you experience an effect on your sleep quality after surgery? Yes or No. If patients reported a positive outcome, they were asked to supplement the answer with a visual analog scale (VAS) in which their agreement of surgical effect was graded in a continuous scale ranging from 0 = no agreement to 10 = full agreement. Scores between 0-3 were defined as “mild”, scores >3-7 were defined as “moderate”, and scores >7-10 were considered “good” [7]. The primary outcome was alterations in the AHI, oxygen desaturation index (ODI), body mass index (BMI) and Epworth Sleepiness Scale (ESS) in the two groups. The secondary outcome was to evaluate the effect of surgery on sleep quality and nasal obstruction reported in the questionnaire. SPSS 19.0 was used for the statistical evaluations. Preoperative and postoperative values were evaluated using the Wilcoxon matched-pairs test in continuous variables without normal distribution (ODI, ESS). Variables with normal distribution (BMI, AHI) were evaluated using the paired t-test. The values for AHI were transformed using natural logarithm in order to create a normal distribution. An independent t-test was used to compare the changes of the objective measures and VAS after surgery between group 1 and 2. Differences with p <0.05 were considered significant.

## Results

**Figure 1:**
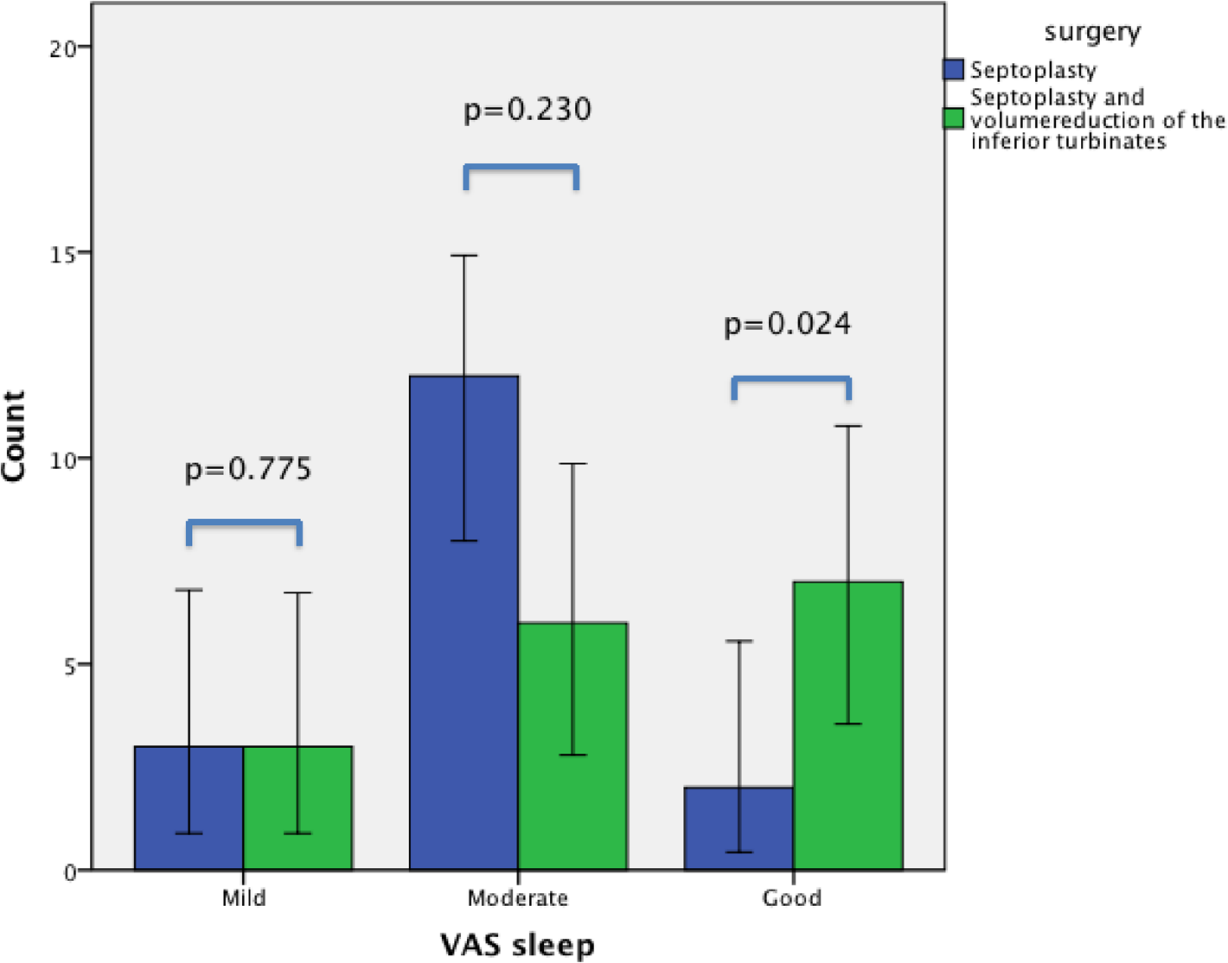
**The self-reported improvement of sleep quality after surgery.** The improvement (VAS sleep) described as mild, moderate or good. The values for septoplasty in blue (left) and the values for septoplasty and volumereduction of the inferior turbinates in green (right).

In both groups, there was a predominance of males (97% in group 1 and 85% in group 2), and the mean age was 47.5 (30 – 68) in group 1 and 45.3 (23 - 68) in group 2. The groups did not differ significantly regarding preoperative AHI, ODI, ESS, Mallampati score, age, gender or BMI. We looked at changes in the objective parameters before and after surgery in three ways: the overall changes in both groups pooled together, changes within each group, and the changes in the mean difference between the groups (Table 1). Overall, in both groups together, there was no significant reduction in mean AHI after surgery: 18.1 (±13.7) - 16.6 (±12.9), (95% CI -1.84, 4.83), p = 0.365, mean ODI: 14.2 (±12.3) – 12.4 (±10.7), (95% CI -1.16, 4.75), p = 0.229 or mean BMI: 28.1 (±3.2) – 28.3 (±3.0), (95% CI – 0.673, 0.285), p = 0.422. The reduction in mean ESS, however, was highly statistically significant: 10.7 (±3.7) – 8.9 (±3.8), (CI 1.00, 2.61), p <0.001. In comparison, when we looked at each group separately, we found a significant reduction in group 2 in mean AHI: 17.4 (±14.4) – 11.7 (±8.2), (95% CI 0.004, 0.006), p = 0.007 and mean ESS: 9.7 (±3.4) – 7.6 (±2.2), (95% CI 0.004, 0.006), p = 0.006. In group 1 there was no significant reduction in mean AHI, ODI or BMI after surgery, but there was a significant reduction in the mean ESS score: 11.5 (±3.7) – 10.0 (±4.5), (95% CI 0.53, 2.54), p = 0.004. The changes in mean ODI levels did not fall below the 0.05 level of significance in either category, although there were near significant values in group 2. The reduction in the difference of mean AHI after surgery was significant between the groups: 1,7 (±8,8) – 5,7 (±16,1), (95% CI 0.8, 14.0), p = 0.029, but the effects on ESS, ODI and BMI were not significant between the two groups . Success criteria defined as a postoperative drop in AHI <20 and/or 50% reduction in AHI [8] were met by 15.2% (5/33) in group 1, and by 27% (7/26) in group 2, but the difference in surgical success was not statistically significant. There were 76% questionnaire responders in group 1 and 77% in group 2. In group 1, 96% answered that the procedure was effective with regard to nasal obstruction, and 68% that it improved their quality of sleep. In group 2 the corresponding percentages were 85% and 80%. The difference between the groups was not statistically significant. A significantly larger proportion in group 2 reported a good improvement in sleep quality: mean 0.08 (±0.27) – mean 0.35 (±0.49), (95% CI 0.037, 0.503), p = 0.024 (Figure 1).

**Table 1:** Baseline values and postoperative values

	**Surgery**					
	**Septoplasty**		**Septoplasty and volume reduction**		**Overall results**	
**Preoperative values**	**Mean**	**SD**	**Mean**	**SD**	**Mean**	**SD**
AHI	18.75	13.36	17.39	14.38	18.15	13.71
ODI	14.29	12.00	14.12	12.73	14.21	12.22
ESS	11.54	3.72	9.74	3.42	10.74	3.67
BMI	28.33	3.40	27.80	3.05	28.10	3.23
Postoperative values	Mean	SD	Mean	SD	Mean	SD
AHI	20.46	14.64	11.70	8.19	16.60	12.90
ODI	14.87	12.25	9.30	7.36	12.42	10.67
ESS	10.00	4.51	7.59	2.18	8.94	3.84
BMI	28.69	3.12	27.77	2.70	28.28	2.95
P-values of the difference						
AHI	0.273		0.007		0.365	
ODI	0.671		0.064		0.229	
ESS	0.004		0.006		<0.001	
BMI	0.202		0.716		0.422	
P-values of the difference between treatment groups						
AHI	0.029					
ODI	0.069					
ESS	0.454					
BMI	0.429					

There are no significant differences at baseline between the groups. There is a significant reduction of AHI between the two surgery groups.

## Discussion

Intranasal surgery is currently regarded as important in order to improve compliance with treatment using nasal continuous positive airway pressure (CPAP)/bilevel positive airway pressure (BiPAP) devices in patients with OSA. The impact of intranasal surgery on objective measurements in OSA patients is unclear, but is regarded as limited, as shown by Verse et al in 2002 [9]. In a rare blinded randomized controlled study with sham surgery (septal resection +/- turbinectomies) in 2008, Koutserelakis et al [10] found responders only in the real surgery group. They concluded that nasal surgery rarely treats OSA effectively. In a meta-analysis of 13 studies that dealt with nasal surgery alone in OSA patients [11], the reviewers concluded that nasal surgery for obstruction alone does not reduce AHI significantly but ameliorates daytime sleepiness and clinical symptoms of snoring. Only one of these studies described a statistically significant reduction in AHI [12]. However, the observation period in this study was only 1 month as opposed to 3 months in ours, and the study group was mixed and underwent either septal resection alone or combined with turbinate surgery. One study by Li et al [13] described a homogenous patient group comparable to ours with septal deviation and hypertrophic inferior turbinates (n = 44). They found no significant effect of surgery on AHI, and a lower success rate of 16%. The procedure differed somewhat from ours in that only septal resections were performed under local anesthesia. It may indicate that the impact of the septal deviation on nasal obstruction preoperatively or postoperatively differs from that in our study. In surgical practice different nasal procedures are often performed simultaneously, and previous clinical studies represent no exception [9,10,12,14,15]. If we had presented the results pooled as a single study group, without a comparison of the two different surgical approaches, we would have missed the statistically significant improvement in patients with combined surgical treatment. Assessments of the overall effect of nasal surgery on OSA predict that 16.7% will have a reduction in AHI [10] that meets the criteria by Sher [8,9]. In this observational study, we singled out two different intranasal surgical procedures for comparison and found that there were statistical differences in the outcome of AHI between septoplasty alone and septoplasty combined with volume reductive surgery in OSA patients. Using the same Sher criteria, we found a near twofold increase in treatment success in the combined surgery group compared with the septoplasty group. This difference did not reach statistical significance but it is possible that it would do so in a larger study group as the difference in AHI reduction was significant. One might anticipate that the better effect on OSA might be due to a larger effect on nasal obstruction in patients in need of combined surgery. It is also possible that the additional inferior turbinate hypertrophy affected the laminar airflow and pharyngeal walls negatively to a higher degree, and hence this group achieved a better result after surgery. Li et al [13] found that patients with a low Friedman tongue position had better results from nasal surgery and Morinaga et al reported less effect in patients with a narrow retroglossal space and high Mallampati score. It may indicate that the increased contribution of pharyngeal structures to OSA will worsen the final results as the percentage of the nasal obstruction is diminished. On the other hand, it may also indicate that the effect of surgery was better for patients with concomitant increased volume of the turbinates and septal deviation because the total contribution of the nasal obstruction to OSA development may have been greater than in patients with septal deviation alone.

In this observational study, there are some limitations that should be taken into consideration. The number of patients in group 2 is low and could represent a statistical uncertainty. There is a higher night-to-night sleep polygraph variation regarding AHI in mild or moderate sleep apnea than in severe apnea that may influence the results on an individual basis [16]. This might suggest that a follow-up study should be performed in patients for whom there is a discrepancy between subjective and objective results. Furthermore, there is a lack of objective measuring of nasal obstruction in an outpatient setting that would otherwise help the surgeon in deciding which type of surgery to perform. Our study is an observational cohort study, and the patients were therefore not randomized to specific treatment groups. As a result, we cannot conclude that combined surgery is better than septoplasty alone in all patients with clinical indications for septal surgery. There may also be possible side effects of supplementing volume reductive surgery in all OSA patients with septal deformities, and this approach should be avoided. However, the results for OSA in our material seemed to be better when both turbinate hypertrophy and septal deviation were treated. Even though combined surgery does not imply a cure for the majority of the patients, there was a reduction of symptoms, verified by the questionnaire, which indicates that 80% perceived an improvement in their quality of sleep after the combined surgery. This study then supports the view that an effect on daytime sleepiness is observed more often than on obstructive apnea and hence that nasal surgery alone is best suited for patients with mild or moderate obstructive sleep apnea. As long as we do not have any single treatment that provides a cure for OSA and not all patients with mild and moderate OSA will accept or tolerate CPAP or oral devices, there will be a place for targeted surgical treatments that improve QOL in these patients.

## Conclusion

In this observational cohort study, the effect on AHI was significantly better when indication for septoplasty combined with surgery of the inferior turbinates was present, compared to septoplasty alone. The overall effect in both groups pooled together showed no significant effect on reduction of the objective parameters but a significant reduction in the subjective ESS score. This implies that intranasal surgery has a good effect on the subjective quality of sleep in OSA patients, and that there might be an added effect on AHI in selected patients with both septal deviation and hypertrophy of the inferior turbinates. Future randomized and prospective studies that can identify responders to nasal surgery as well as what type of intranasal surgery needed.

## Competing interests

The authors declare that they have no competing interests, neither financially nor non-financially.

## Authors’ contributions

MM performed the surgery, collected the sleep related perameters, analysed data and contributed in writing the manuscript. SN contributed to design, analysed and interpreted data, contributed in writing the manuscript, and reviewed the final version. Both authors read and approved the final manuscript.

## Pre-publication history

The pre-publication history for this paper can be accessed here:

http://www.biomedcentral.com/1472-6815/14/11/prepub
